# A Central Spiral Split Rectangular-Shaped Metamaterial Absorber Surrounded by Polarization-Insensitive Ring Resonator for S-Band Applications

**DOI:** 10.3390/ma16031172

**Published:** 2023-01-30

**Authors:** Shihabun Sakib, Ahasanul Hoque, Sharul Kamal Bin Abdul Rahim, Mandeep Singh, Norsuzlin Mohd Sahar, Md. Shabiul Islam, Mohamed S. Soliman, Mohammad Tariqul Islam

**Affiliations:** 1 Institute of Climate Change, Universiti Kebangsaan Malaysia, Bangi 43600, Selangor, Malaysia; 2 Wireless Communication Centre, Universiti Teknologi Malaysia, Skudai 81310, Johor, Malaysia; 3 Faculty of Engineering (FoE), Multimedia University, Persiaran Multimedia, Cyberjaya 63100, Selangor, Malaysia; 4 Department of Electrical Engineering, Collage of Engineering, Taif University, P.O. Box 11099, Taif 21944, Saudi Arabia; 5 Department of Electrical Engineering, Faculty of Energy Engineering, Aswan University, Aswan 81528, Egypt; 6 Department of Electrical, Electronic and Systems Engineering, Faculty of Engineering and Built Environment, Universiti Kebangsaan Malaysia, Bangi 43600, Selangor, Malaysia

**Keywords:** metamaterial absorber, spiral split, polarization, low frequency

## Abstract

This paper reports a central spiral split-rectangular-shaped metamaterial absorber surrounded by a polarization-insensitive ring resonator for s-band applications. The rated absorption is 99.9% at 3.1 GHz when using a three-layer structure where the top and ground are made of copper and the center dielectric material is a commonly used FR-4 substrate. The central split gaps have an impact on the unit cell by increasing high absorption, and an adequate electric field is apparent in the outer split ring gap. At 3.1 GHz, the permittivity and permeability are negative and positive, respectively, so the proposed unit cell acts as an epsilon negative (ENG) metamaterial absorber. In a further analysis, Roger4450B was used as a substrate and obtained excellent absorption rates of 99.382%, 99.383%, 99.91%, and 95.17% at 1.44, 3.96, 4.205, and 5.025 GHz, respectively, in the S- and C-band regions. This unit cell acts as a single negative metamaterial (SNG) absorber at all resonance frequencies. The S_11_ and S_21_ parameters for FR-4 and Rogers4450B were simulated while keeping the polarization angle (θ and φ) at 15, 30, 45, 60, 75, and 90 degrees to measure, permittivity, permeability, reflective index, absorption, and reflection. The values of the reflective index are near zero. Near-zero reflective indexes (NZRI) are widely used in antenna gain propagation. The unit cell fabricated for the FR-4 substrate attained 99.9% absorption. S-band values in the range of (2–4) GHz can be applied for low-frequency radar detection.

## 1. Introduction

Complex research into super lenses, antenna designs, image sensing, low-cross-section materials for radar, and absorber technologies requires a material that shows negative permittivity, permeability, and reflective index properties. However, naturally available materials do not exhibit such characteristics. Unlike natural materials, metamaterials are constructed artificially with unique electromagnetic (EM) properties. Metamaterials can be used for operations at desired frequencies. A super lens constructed from a metamaterial that exhibits negative unity permeability with a 35.3 mm wavelength at 8.5 GHz has been proposed by Scarborough [1]. A lens close to 23 cm square and 4 cm thick was designed to construct a functional prototype. Metamaterial-based antennas [2] are a very attractive research area for scientists. Planer antennas are widely used for wideband [3] and ultra-wideband [4,5,6,7,8,9] (UWB) applications. UWB antenna design aims to create a tiny and simple antenna with low distortion and a large, wide bandwidth. These antennas are mostly constructed from inexpensive FR-4 substrate with copper patches. The rare features of MM-based imaging technology have created immense interest in many areas of current research, such as brain stock imaging [10]. Furthermore, MM is used for dual-band [11], triple-band [12], flexible broadband [13], and wideband [14] absorbers with polarization insensitivity [15]. However, naturally available materials do not exhibit such characteristics. Unlike natural materials, metamaterials are constructed artificially with unique electromagnetic (EM) properties. Metamaterials (MMs) can be used for operations at desired frequencies. Due to their stunning properties, MMs are widely used in many research fields. An important characteristic of MMs is their absorption of EM waves. This behavior means that metamaterial absorbers (MAs) have become well-known. When EM waves pass through MAs, they experience a significant attenuation. Due to this mechanism, MAs are used in many research fields.

The concept of a metamaterial absorber has become popular and has garnered much attention from researchers since 2006, when M.A. Bilotti et al. [16] first invented an MA based on a split-ring resonator. This design attracted much interest among scientists because it could absorb resonance microwaves. Usually, the dielectric substrate of an MA is covered by copper from both sides (top and bottom). A polarization-independent MM absorber has been proposed for multiple band applications, including C, X, and Ku [17]. Another special characteristic of MM absorbers is their zero-reflective index property (NZI). This property occurs when the ratio of permittivity and permeability becomes zero. NZI surfaces can absorb EM waves. Due to this absorption, NZI surfaces are applicable to enhance antenna gain [18] and the directivity of antenna radiation [19,20,21,22,23,24]. Controlling directivity is necessary for the stable communication of high-frequency antennas. Nowadays, absorbers are used in the photovoltaic region, such as the solar-photovoltaic and thermo-photovoltaic bands [25,26]. K- and Ku-band sensing devices have been constructed using MAs in recent years [27,28].

Although there are multiple applications of MAs, many still do not have 99.9% absorption in the S-band. For example, a Jerusalem cross [29] MA has been designed with 95% absorption. Ahasanul proposed a pitch-square-shaped MA with 99.9% absorption, but it was not applicable for S-band applications [30]. Dielectric materials have a vital effect on frequency selective surfaces (FSSs), such as switchable passband screens [31] and broadband radar absorbers [32]. It was found in [33] that the resonance frequency of FSSs without any dielectric is 6.32 GHz, whereas with an FR-4 substrate it is 3.81 GHz. The term “FSS” denotes metallic or dielectric elements arranged in a periodic pattern to create a spatial filtering effect. These papers were researched based on various excitation angles, but the proposed designs were analyzed based on various polarization angles. Previously reported S-band absorbers, including modified C-shaped [34], reformed I-shaped [35], and double-C-shaped [36] metamaterials, cannot be used as absorbers. S-band absorption is relevant to our proposed MA. This makes our design different from other proposed S-band metamaterials.

In this article, an excellent MM absorber is constructed by plotting the resonator sequentially on both sides of the substrate. We develop a miniature metamaterial absorber with orthogonal phases and maximum field concentration properties that can be used for S-band applications. The central split spiral rectangular-shaped structure in the unit cell increases the maximum absorption rate up to 99.9%. The proposed cell depicts the maximum absorption at 3.1 GHz, and Rogers4450B is used as a substrate and shows excellent absorption in the S- and C-bands at 1.44, 3.96, 4.205, and 5.025 GHz. The designed cell is analyzed with Rogers4450B and FR-4 substrates at different polarization angles for the electric and magnetic fields. Finally, 1 × 2 and 2 × 2 arrays are designed, and the absorption rates are 96.8% and 95.7% at 3.09 GHz and 3.085 GHz, respectively. The experimental design is easy to construct and flawless in terms of its S-band applications.

## 2. Design and Methodology

**Figure 1:**
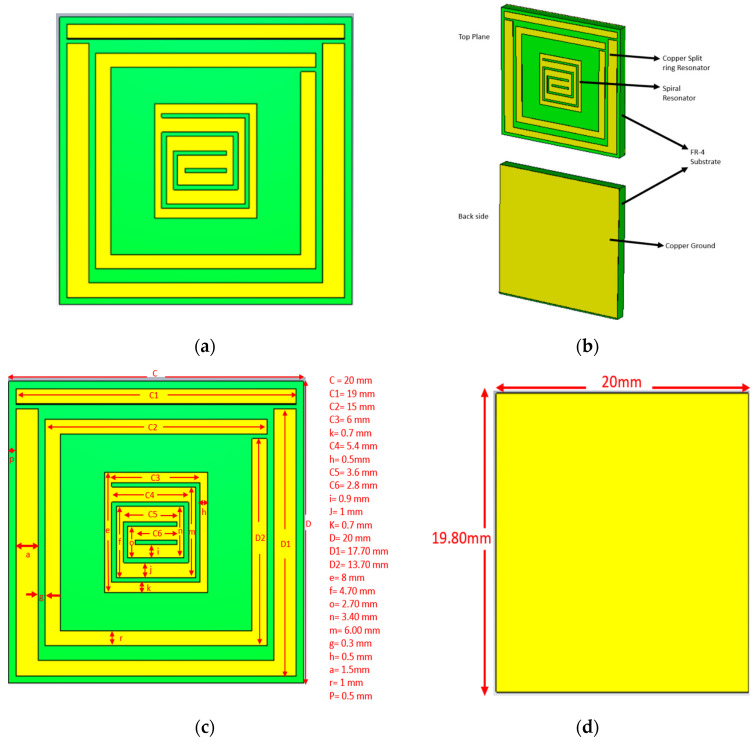
The proposed unit cell. (**a**) Split-ring resonator with a rectangular snake-cutting structure. (**b**) The complete construction of the MA. (**c**) All of the copper parameters. (**d**) The ground.

**Figure 2:**
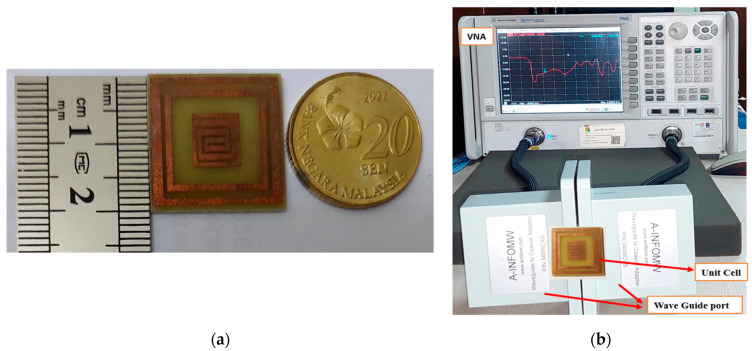
The designed unit cell. (**a**) Fabricated. (**b**) Measured by VNA.

**Figure 3:**
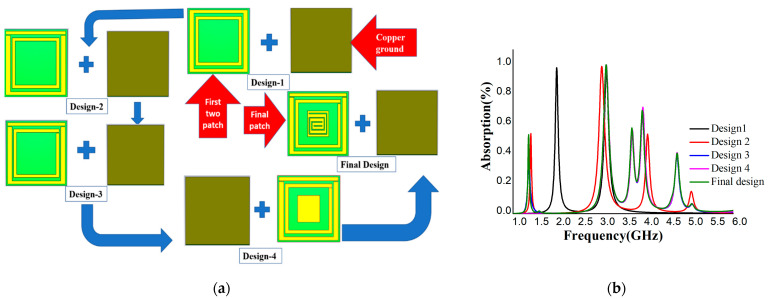
(**a**) Final design steps for unit cell. (**b**) Absorbance at all steps.

**Figure 4:**
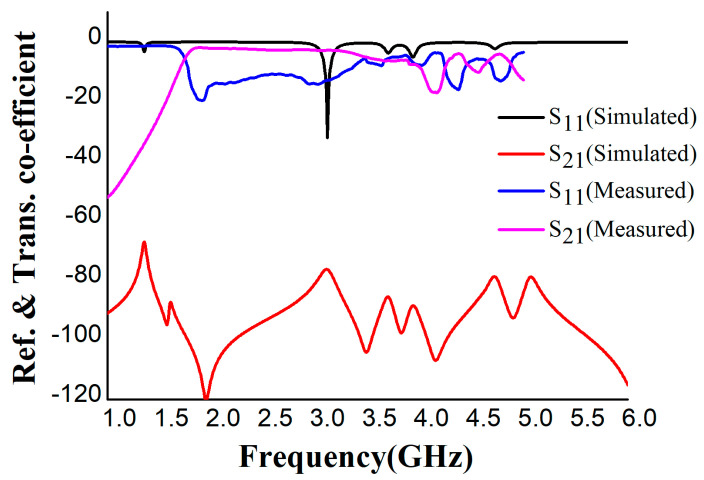
Reflection (S_11_) and transmission (S_21_) co-efficient performance at the dB scale.

The unit cell was designed to give a perfect metamaterial absorber at the resonance frequency. Commercially available FR-4 substrate with a 1.6 mm thickness was chosen for the absorber. The top plane and the ground of the unit cell were constructed with 0.035 mm copper (annealed), and copper covered both sides of the substrate. The proposed resonator has two spilt-ring resonators enclosing a rectangular snake-cutting structure (Figure 1a), and the central square acts as a spiral split-a-shape resonator. The unit cell was also analyzed at different polarization angles, including 15°, 30°, 45°, 60°, 75°, and 90° for the electric and magnetic fields. The polarization angles θ and φ were moved to the E-field and H-field directions. The whole patch structure is manipulated in such a way that all the EM waves must be absorbed. The proposed MA was fabricated and measured. Figure 2a is the fabrication of the unit cell, and Figure 2b in the manuscript illustrates the experimental setup for analyzing the S_11_ and S_21_ of the proposed absorber between the two waveguide ports. We used a the Keysight N4692A MW electronic calibrator (Ecal). After Ecal calibration, W/G N4697-60200 coaxial cables with C8155 adapters were connected with P/N:340WCAS(A-INFOMW) waveguide ports over the range of 2.2 to 3.95 GHz so the wave could interact with the proposed unit cell perfectly. The complete set-up was connected to a vector network analyzer (VNA) to measure the S-parameters. During the measurement, we first set the frequency range of 1–4 GHz in VNA as the waveguide port frequency range. To reduce the noise, we filtered out the additional noise using a smoothing option up to 4%. Then, we held the measured result and took pictures. Finally, the files were saved in .png form and the data were extracted using MATLAB software. The center gaps increase the absorption rate, which is visible in Figure 3b in the final design. The absorption value and S-parameter (S_11_) are 98% and −27.8 dB, respectively, when the 0.3 mm central gaps are missing. These values are increased to 99.9% at −32.2 dB with a 0.3 mm gaps in the center. The simulated and measured values of S_11_ and S_21_ are shown in Figure 4.

The proposed unit cell’s two resonators have three 0.3 mm gaps that enhance the electric field, and the entire spit ring can be formed by copying at 90°, 180°, and 270° shifts. The 0.3 mm gap rotates circularly and ends at the center. The complete construction of the unit cell can be easily understood from Figure 1b. Figure 1c,d illustrate the copper parameter for the proposed design. The back side of the whole substrate surface area (20 mm × 20 mm) is covered by ground. The ground is cut at the end of the area (20 mm × 0.2 mm). The commercially available computer simulation software CST-2019 is used to conduct high-frequency numerical analysis. CST has an excellent finite integration technique that can determine reflection, transmission, and absorption. A perfect EM wave can be obtained along the Z-axis by applying an ideal electric and magnetic field along the X-axis and Y-axis, respectively.

## 3. Theory of Absorption

As the ground of the unit cell is copper, the transmission and reflection of the EM waves may become weak when they pass through the MM structure. Thus, the waves are absorbed by the structure. The absorption can be calculated from:

(1) $$A\left(\omega \right)=1-R\left(\omega \right)-T\left(\omega \right)$$

From Equation (1), by calculating the reflection co-efficient **R**(**ω**) and transmission co-efficient **T**(**ω**), the proposed metamaterial absorbance can be obtained. **R**(**ω**) and **T**(**ω**) can be represented by **R**(**ω**) = |**S**_11_|^2^ and **T**(**ω**) = |**S**_21_|^2^, where S_11_ is the reflectance wave from the unit cell to port 1 and S_21_ is the transmittance through the cell from port 2 to port 1. To extract the primary parameters (S_11_, S_21_), Smith et al. [37] proposed an S-parameter method. S-parameters can be represented by:

(2) $$S_{11}=\frac{R_{H}\left(1-e^{i2nk_{0}d}\right)}{1-R_{H}^{\;2}e^{i2nk_{0}t}}$$

(3) $$S_{21}=\frac{\left(1-R_{H}^{\;2}\right)\;e^{ink_{0}d}}{1-R_{H}^{\;2}e^{i2nk_{0}d}}$$

where **R_H_** = (**z** − **1**)/(**z** + **1**), which can calculate the reflection coefficient for a half-space; **z** is the impedance of the MM unit cell medium; **η** is the reflective index; and **d** is the substrate thickness.

Here,

(4) $$e^{i2nk_{0}t}=p\pm i\sqrt{1-p^{2}}$$

and

(5) $$Z=\sqrt{\frac{{\left(1+S_{11}\right)}^{2}-S_{21}^{2}}{{\left(1+S_{11}\right)}^{2\;}-S_{21}^{2}\;}}$$

where $p=\left(1-S_{11}^{2}+S_{12}^{2}\right)$. The absorber’s permittivity and permeability can be calculated by $\varepsilon =\frac{\eta }{z}$ and μ=ηz. From Equations (3) and (4), the reflective index **η** can be calculated as follows:

(6) $$\eta =\frac{1}{k_{0}t}\cos^{-1}\left[\frac{1}{2S_{21}}p\right]$$

The proposed unit cell’s impedance, reflective index, and absorption capacity can all be calculated easily from Equations (5) and (6). According to Equations (2) and (3), all the parameters rely on S_11_ and S_21_.

In the proposed design, the ground is covered by copper. Therefore, the skin depth equation can be analyzed from the perspective of the electromagnetic wave blocking the unit cell from port 2 to port 1.

(7) $$\delta =\sqrt{\frac{\rho }{\pi f\mu }}$$

Here, in Equation (7), resistivity **ρ** = 1.72e − 8 Ω-m, conductivity **σ** = 5.8e + 7 s/m, and permeability **µ** = 1. At 3.1 GHz resonance frequency, the value of Equation (7) is **δ** = 0.001186 mm. The skin depth result explains that the S_21_ transmission of the EM wave is completely interrupted from port 2 to 1 by copper (annealed). Equation (1) in this manuscript can be rewritten as:

**A = 1 − R(ω)** (8)

However, there is no direct relation between the MM’s absorption and the negative reflective index [38] at resonance frequencies. An MM that has both absorptivity and negative refractive index can be added as an advantage, leading to many guided wave applications in the microwave range [39]. In a perfect MM absorber, to prevent EM energy dissipation from the absorber, the conservation of energy laws’ should be applied following the complex Poynting vector [40]. As a result, the MM does not act as a transient medium and absorbs the desired EM energy frequencies. The permittivity and the reflective index values must be real [41] and negative to conserve the energy that leads to the MM’s characteristics, which can be set accordingly at different resonance frequencies. These technical aspects cover many vital performances parameters for some applications such as radar cross-section reduction, EM stealth mood, sensing, etc. [42], where MM properties and EM energy conservation are needed for the required performance.

## 4. Characterization of the Absorber

The unit cell must be designed in a way whereby it shows high absorption in the S-band. For this purpose, the resonators and the rectangular snake-cutting-shaped structure must include 0.3 mm gaps. These gaps create the capacitive property. The ground that is covered with copper is cut at the edge for excellent absorption [43] with a negative reflective index, as described in Equation (6). The total areas for the two split-ring resonators are 73.4 mm^2^ and 58.7 mm^2^. Additionally, the center rectangular-shaped structure has an area of 30 mm^2^. The two split-ring resonators and a center rectangular-shaped structure are constructed sequentially to ensure the highest absorption. There are five different methods, as shown in Figure 3a, of achieving high absorption. The absorption is highest (99.9%) for the final design. Another resonance can be produced with the capacitive element around the unit cell’s circumference. Hence, five distinct absorption peaks are obtained, as shown in Figure 3b. The shifted resonance frequencies are achieved by cutting the edge of the ground. This can be seen in Figure 3a, where the central rectangular-shaped copper split is cut in the shape of a snake with 0.3 mm gaps. The gap in the center creates more absorption than the other four designs.

The reflection (S_11_) and transmission (S_21_) co-efficient performance at the dB scale is shown in Figure 4. There are differences between the measured and simulated results of the S_21_ parameter. When we attached the waveguide ports at the time of calibration using a Keysight N4692A MW electronic calibrator (Ecal), some air interference may have affected the propagation from the excitation port to the unit cell, which may have triggered performance variations. Another possible reason for the result deviation could be the lower concentration of copper in the layer at the edge of the ring resonator during fabrication. A 0.01 mm copper layer on the banding area of the ring resonator of the unit cell is missing. We measured the S_21_ at different gap variations between the unit cell and waveguide ports to assess the mutual resonant effects of the waveguide ports. At 0.1 mm distance, the S_21_ is −40 dB at 4.3 GHz, but the noise concentration is high. When the unit cell is in the optimized position concerning the waveguide ports, Figure 4 shows the final effect with less noise.

## 5. Analysis of Absorber

**Figure 5:**
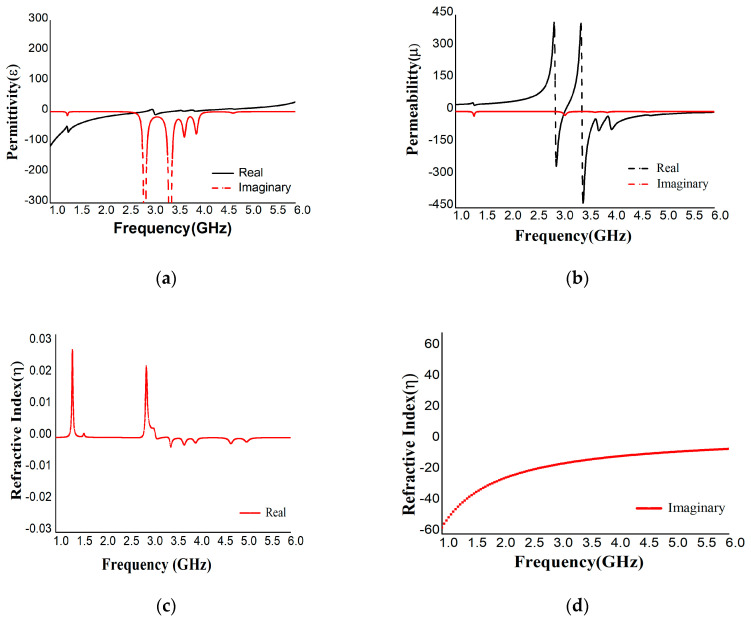
Real and Imaginary values of (**a**) permittivity, (**b**) permeability, and (**c**,**d**) reflective index for FR-4 substrate.

The proposed MA was designed to absorb microwave frequencies successfully and efficiently in the S-band range. A rectangular-shaped structure surrounded by two split-ring designs was selected for the highest absorption. The central rectangular structure has a 0.3 mm snake-shaped gap, and the two resonators have three 0.3 mm gaps. Solid copper was used on the opposite surface of the resonator as a ground to block the transmittance and reflectance waves in the expected frequency ranges with a refractive index near zero or negative. After simulation in CST, the real and imaginary values of S_11_ and S_21_ were exported to excel and then plotted in MATLAB to obtain the perfect values of permeability, permittivity, reflective index, and absorption shown in Figure 5a–c and Figure 3b.

The maximum absorption of 99.94% was achieved at 3.1 GHz frequency with negative permittivity and positive permeability with a 4.6 dielectric constant FR-4 substrate. As the proposed unit cell has negative permittivity and positive permeability, it can be applied as an ENG absorber. The incident waves at the different polarization magnetic and electric angles (0°, 15°, 30°, 45°,60°, 75°, and 90°) were also analyzed to obtain permittivity, permeability, and reflective index values, as shown in Table A1 in Appendix A for the S_11_ parameter. The permittivity is always negative for the polarization angle φ and most values are positive for the polarization angle θ, and vice versa for the permeability. The lowest value (0.06) of reflection was observed at 3.1 GHz, and the reflective indexes were near zero. MATLAB was also used to extract S_21_ values for polarization angles (θ, φ) 0°, 15°, 30°, 45°, 60°, 75°, and 90°, as listed in Table A1. The reflection was 99.68% at 1.95 GHz, which was the highest value; at the same time, the absorption is 0.3%, which is the lowest value. From Table A1, the absorption value is at its maximum at the S_11_ resonance frequency and at its minimum at the S_21_ resonance frequency.

**Figure 6:**
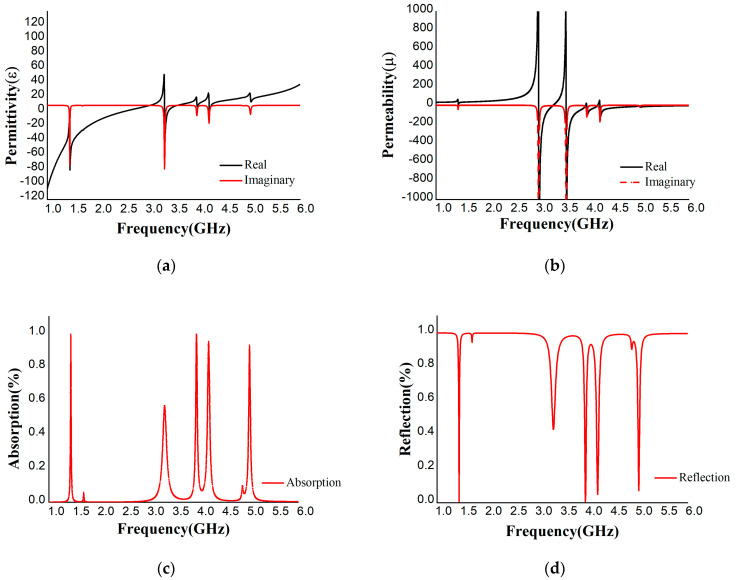
(**a**) Permittivity, (**b**) permeability, (**c**) absorption, and (**d**) reflection of Rogers4450B substrate.

The proposed absorber was also simulated with a Roger4450B (dielectric constant = 3.7, thickness = 1.575) substrate. The variations in permittivity, permeability, and reflective index with respect to polarization angle are listed in Table A2 in Appendix A and displayed in Figure 6a–d. The S_11_ and S_21_ parameters were extracted from MATLAB. Table A2 shows four resonance frequencies at 1.44, 3.96, 4.205, and 5.025 GHz with 99.382%, 99.383%, 92.91%, and 95.17% absorption, respectively. The permittivity is positive at 3.96 GHz and 5.025 GHz and negative at 1.94 GHz and 4.205 GHz and vice versa for permeability. Hence, the design is an excellent absorber with SNG properties in the S- and C-band regions. From the numerical analysis, it can be concluded that FR-4 has a wider band (3.09–3.14 GHz) than Roger4450B and a high absorption of 99.94% and −32.15 dB for S_11_, whereas Roger4450 exhibited values of 99.38% and −22.10 dB for S_11_. Moreover, the dielectric constant, loss tangent, and thermal conductivity of FR-4 were 4.3, 0.025, and 0.3 w/k/m, respectively, whereas Rogers4450B showed values of 3.6, 0.004, and 0.6 w/k/m, respectively. The loss tangent of FR-4 was greater than that of Roger4450B, but the dielectric property we found for FR-4 was comparatively stable compared to that of Roger4450B. Thus, absorption was obtained more for FR-4. We did not achieve the dielectric property that Roger4450B showed during the simulation. In this scenario, the absorption rate was decreased for Rorger4450B.

**Figure 7:**
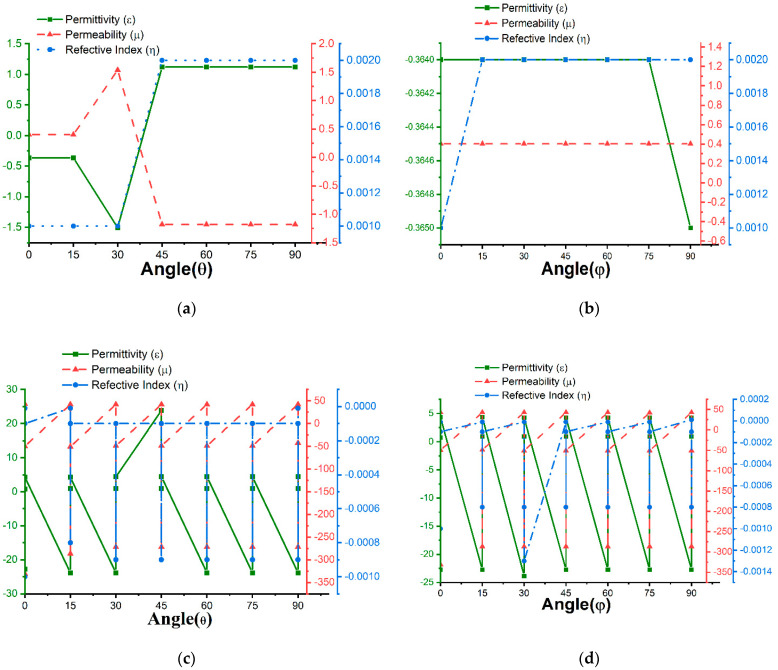
FR-4 permittivity, permeability, and reflective index with respect to polarization angle θ and φ for (**a**,**b**) S_11_ and (**c**,**d**) S_21_.

**Figure 8:**
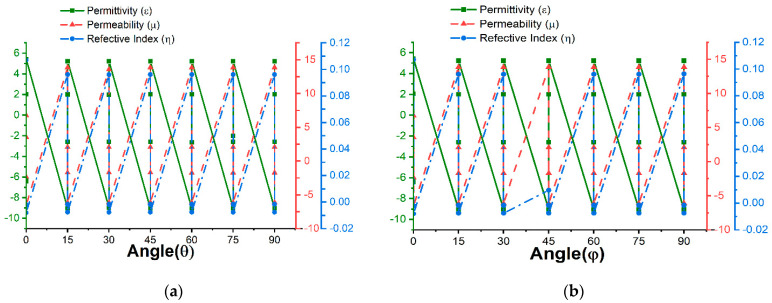

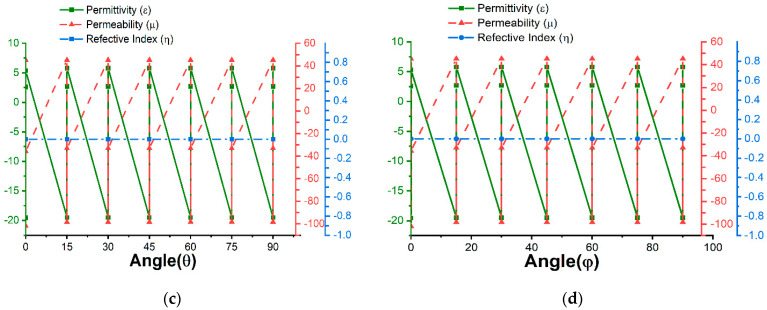
Roger4450B permittivity, permeability, and reflective index with respect to polarization angle θ and φ for (**a**,**b**) S_11_ and (**c**,**d**) S_21_.

The polarization insensitivity can be more visual from Figure 7 and Figure 8. These Figures are the form of Table A1 and Table A2. The permittivity, permeability, and reflective index for FR-4 and Roger4450B are depicted in Figure 7 and Figure 8. The reflective index of the substrates is near-zero for all the polarization angles. The permittivity and permeability values are opposite. When the permittivity is positive, the permeability becomes negative and vice versa. The permittivity, permeability, and reflective index values differ slightly for the polarization angles for 0°,15°,30°,45°,60°,75°, and 90°. Hence, the proposed design is polarization insensitive.

**Figure 9:**
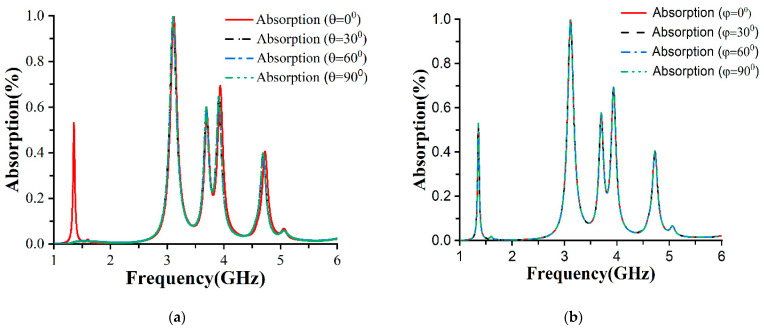
Absorption rate of FR-4 for different polarization angles (**a**) θ and (**b**) φ.

Absorption at the different polarization angles is depicted in Figure 9a,b. The proposed metamaterial absorber (MA) is insensitive to polarization because the absorption curves did not change with 0°, 30°, 60°, and 90° θ and φ rotations. This performance has increased MA eligibility. Typically, polarization insensitivity can be acquired by symmetrical design. The symmetrical patch arrangement of the design is the main mechanism that has a crucial impact on the absorption rate with the angular rotation of the unit cell. Figure 9a,b were plotted for FR-4, and data were collected from Table A1 in Appendix A.

## 6. Analysis of Current and Field Distribution of Unit Cell

**Figure 10:**
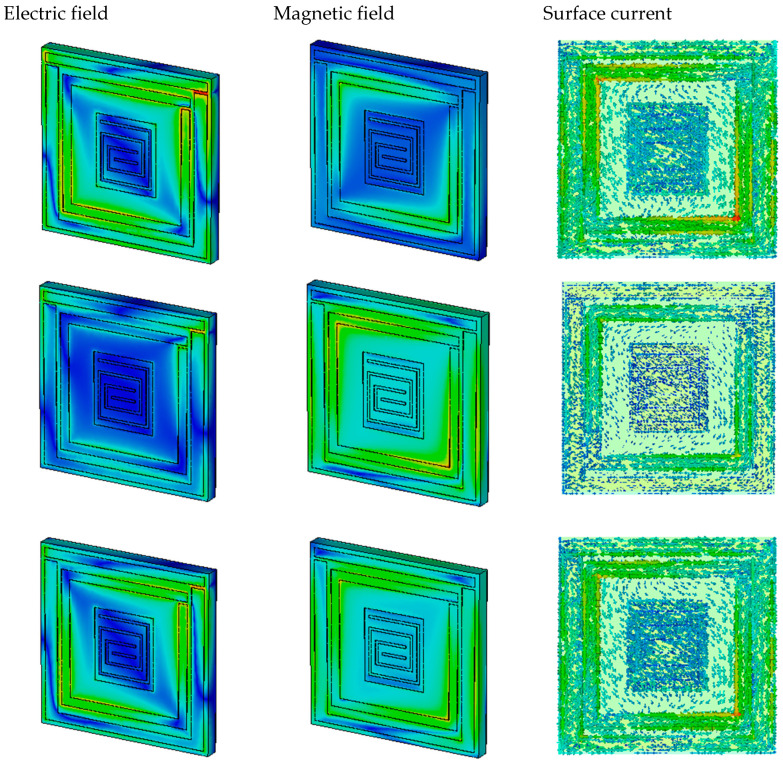

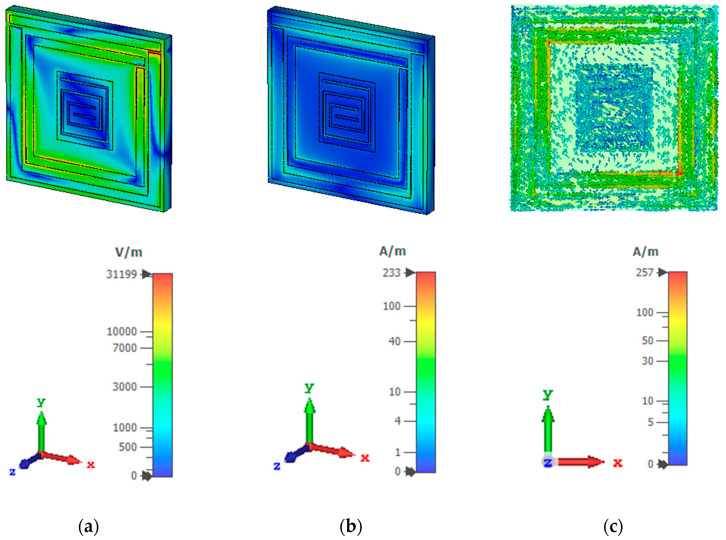
(**a**) Electric field, (**b**) magnetic field, and (**c**) surface current density of FR-4 substrate at 1.95 GHz, 3.49 GHz, 4.155 GHz, and 3.1 GHz.

**Figure 11:**
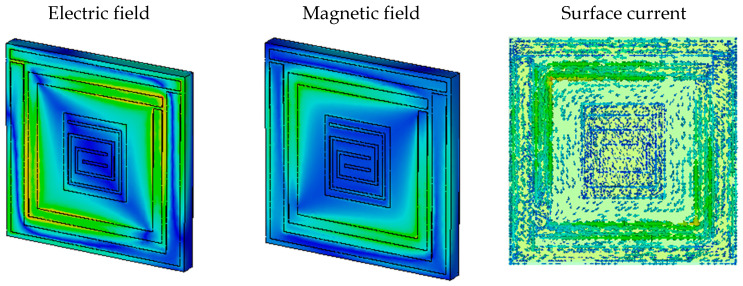

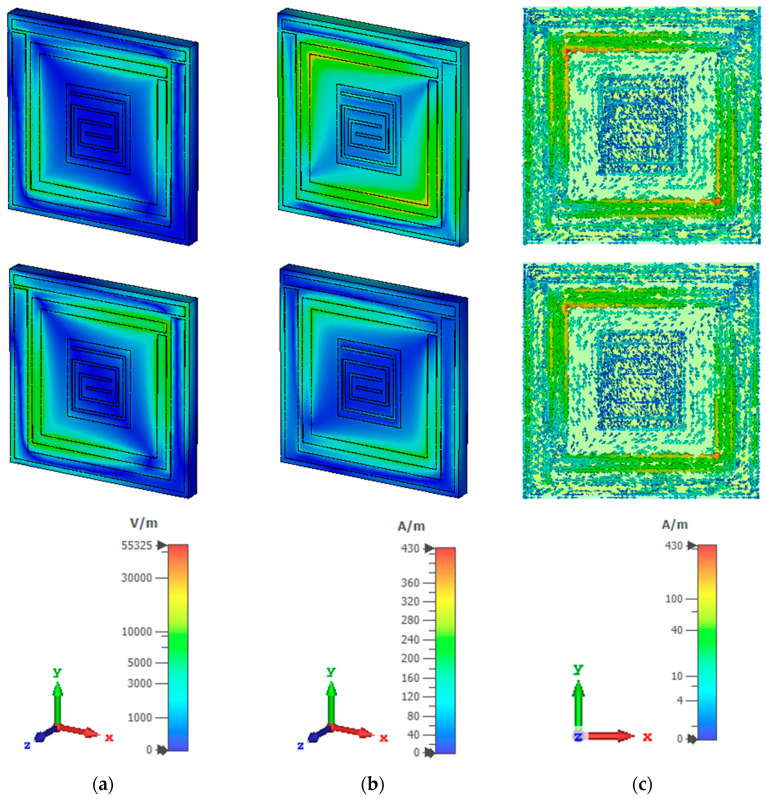
(**a**) Electric field, (**b**) magnetic field, and (**c**) surface current density of Roger4450B.

A clear explanation of the electric field, magnetic field, and surface current distribution can be gleaned from Figure 10 and Figure 11. All the fields are shown at the resonance frequencies. The proposed unit cell exhibits a perpendicular E-field and H-field at four resonance frequencies. The cell was constructed from two substrates, FR-4 and Rogers4450B. The E-field concentration, H-field concentration, and surface current density can clearly be seen for the FR-4 substrate from Figure 10a–c. The concentrations of the E-field and H-field are high at the edge of the patch. At 3.1 GHz, the absorption and the E-field concentration were high. The permittivity, permeability, and reflective index were also measured, and 99.94% was the highest absorption rate for this resonance frequency. At 1.95 GHz, the electric field is high at the outer patch, inner patch, and the red region in the gaps of the patch, but the magnetic field concentration is visible only at the band of the internal split ring. Consequently, the permeability value is higher than the permittivity, and the surface current is less dense than 3.1 GHz. The red region indicates the high-density field concentration. The E-field concentration moves into the inner patch, and the H-field becomes higher for the outer split-ring resonator at 3.49 GHz. At this point, the permittivity is positive and the permeability and reflective index are negative. Gradually, the magnetic field increases and the electric field concentration decreases. The permittivity is positive, and permeability is negative at 4.155 GHz. Therefore, the unit cell exhibits SNG absorption and near-zero reflective indexes at all the resonance frequencies in the S- and C-bands.

For Roger4450B, the electric field, magnetic field, and surface current are demonstrated in Figure 8 at 2.05 GHz, 3.735 GHz, and 4.4 GHz. In Figure 11a–c, we can see that the surface current is high where the electric field is also high, and the magnetic field is less intense in this portion. Therefore, Roger4450B also behaved as an SNG absorber and achieved near-zero reflective indexes similar to FR-4 substrate.

The E-field, H-field, and surface current results show that the electric field concentration is proportional to the surface current density and that magnetic field lags behind the electric field. Maxwell’s equation can explain this property:

(9) $$\nabla \times H=J+\in \frac{\partial E}{\partial t}$$

From Equation (9), the variation between current density and electric field can be explained as:

(10) J=σE

**J** is the current density and **E** is an electric field. The relation between **J** and **E** shows that **J** depends on **E**. The maximum concentration of the electric field in the patch occurs with the highest concentration of the surface current with respect to time. **E** and **J** also follow the H-field direction. Hence, the H-field concentration is sparser than the E-field concentration, which can be seen in Figure 10 and Figure 11.

## 7. Array Analysis

**Figure 12:**
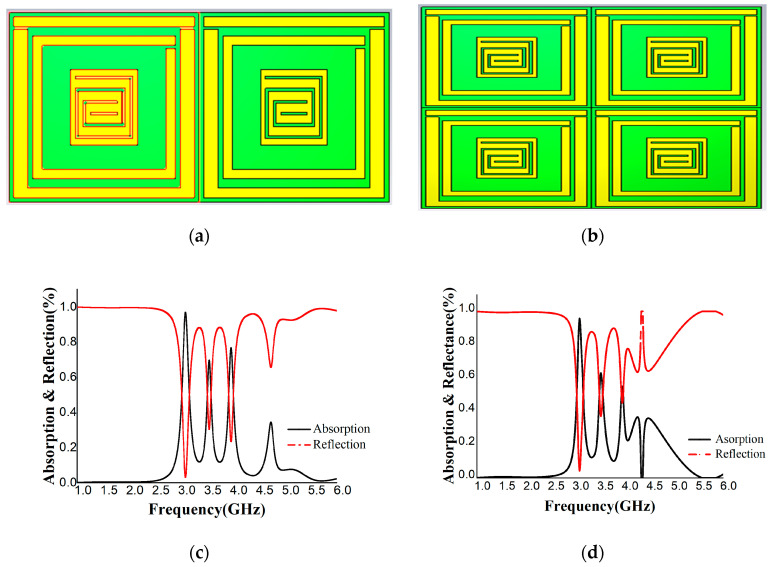
Depiction of the (**a**) 1 × 2 array and (**b**) 2 × 2 array. Absorption and reflection of the (**c**) 1 × 2 array and (**d**) 2 × 2 array.

The proposed unit cell was also analyzed for array structures. Figure 12a,b show the design structure, and Figure 12c,d depict the absorption and reflection percentages. The reflection is 0.3118 and the absorbance is 96.88% at 3.09 GHz for the 1 × 2 array structure. The reflection is 0.0431 and the absorption is 95.69% for the 2 × 2 array structure. In Figure 12c,d, dispersion is present in the array structure but is at a minimum level. Therefore, absorption is decreased here. Another possible scenario is that the mutual coupling patch of the array structure creates the additional capacitance. When we designed the array, the mutual coupling of patch structures formed the additional capacitance, and as a result, a circuitry operation formed. This circuitry operation generated additional absorption. Due to this operation, the absorbance rate decreased.

**Figure 13:**
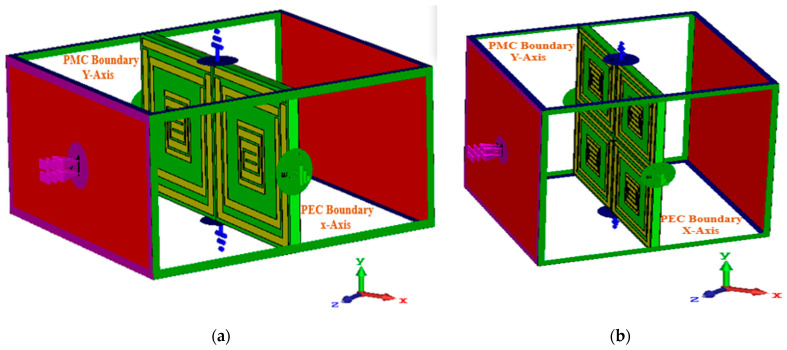
Array analysis. (**a**) 1 × 2 and (**b**) 2 × 2 with boundary conditions, PEC (perfect electric field) PMC (perfect magnetic field).

The performance of unit cells with 1 × 1 and 2 × 2 arrays are shown but not shown for any single-unit cells or for any infinitely periodic cells. Figure 13a,b depict simulated boundary conditions where a perfect electric (PEC) and a perfect magnetic field (PMC) are applied along the X-axis and Y-axis, keeping the Z-axis open.

## 8. Comparison

Around the world, researchers have modified their metamaterial absorber structures to achieve maximum absorption for different applications. For example, In Table 1 L-shaped [44] and T-shaped [45] modified square metamaterial absorbers have been proposed for radar absorption and electromagnetic interference/electromagnetic compatibility (EMI/EMC) configurations. FR-4 substrate was used for the fabrication of these unit cells as it is commercially available and of low cost; however, in terms of absorption, the operating range of these MAs is only 90–96% in the X-band. On the other hand, Borah [46] and Prakakash Ranjan [47] have achieved high absorption above 98% in the X-band. However, they have not shown whether or not their proposed designs are polarization independent. A polarization-independent C-shaped ring MMA has been designed for Ku-band sensing applications, but its absorption level is 99.6%. Our proposed design’s absorption level is 99.94%. It is also polarization-independent and suitable for S-band sensing applications.

## 9. Sensing Application

**Figure 14:**
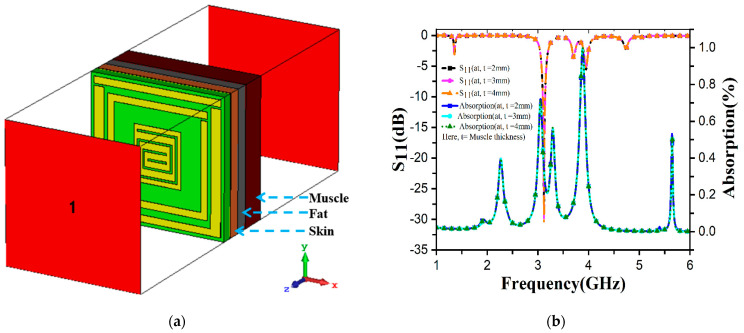
(**a**) Sensing model of FR-4 based MA. (**b**) S_11_ and absorption plotting at different thicknesses of human muscle.

The S-band is designed for low-power microwave equipment, including Bluetooth headphones, Wi-Fi, baby monitors, garage door openers, keyless vehicle locks, and microwave diathermy devices. This band is also used for radar applications to control air traffic, ships, and weather stations. Among these, microwave diathermy (MD) is the specific S-band application area that we are interested in. The therapeutic procedure known as diathermy is most frequently recommended for disorders affecting the muscles and joints. Shortwave, microwave, and ultrasound are the three main types of diathermies. Microwave diathermy (Md) uses electromagnetic radio waves. During diathermy treatment, the heat induced in tissues depends on the conductivity of the tissues involved. Muscle tissues are more conductive than skin and fat tissue. When MD generates heat at the particular area of the human muscle that may suffer from physical problems such as joint pain, deformations, injuries, and so on, the properties of S_11_ in that certain muscle area are also altered. In this case, we can measure changes in the S_11_ values with our unit cell as a passive sensor, and this process is non-invasive. Non-invasive techniques do not employ instruments that penetrate the skin or inside the body and typically do not spread to or harm other tissues and organs. Figure 14a shows the complete simulation setup, where the sensor was installed scientifically with much thought. The thickness, permittivity and permeability of the skin, fat and muscle are listed in Table 2. The ground plane of the metamaterial absorber (MA) was placed a 0.1 mm air gap from the particular human body part, then 2 mm thick skin, fat, and 2/3/4 mm muscle layers were sequentially placed so that the electromagnetic wave from the proposed MA can penetrate properly into the muscle layer. Table 2 demonstrates the permittivity and thermal conductivity values of skin, fat, and muscle. From Figure 14b, when the muscle is 2, 3, and 4 mm, the S_11_ values are −28.7269 dB, −30.189 dB, and −30.359 dB at 3.1GHz, respectively. Absorption levels of 99.8659%, 99.9042%, and 99.9079% were obtained for the three individual muscle layers, respectively. Due to different muscle layers, the absorption curve and S_11_ are changed. The simulation result showed that the values of S_11_ and absorption increased when the muscle thickness increased. This investigation concludes that the sensor can detect deformation, injuries, and other abnormalities in muscle layers by monitoring the S_11_ and absorption values of the specific body parts.

## 10. Conclusions

A single-negative spiral split-rectangular-shaped resonator surrounded by two split-ring copper patches having almost perfect absorption for S-band application was studied in this paper. The effect of the physical structure of the patch on absorption was thoroughly studied, including the central snake cutting gaps. A numerical analysis was conducted in a CST simulator, and the results of absorption, permittivity, permeability, and refractive index were further analyzed using MATLAB. The model achieved similar results in both programs. The absorption rate was 99.94% at 3.1 GHz in the S-band with FR-4 substrate, and 99.382%, 99.383%, 92.91%, and 95.17% at 1.94, 3.96, 4.205, and 5.025 GHz, respectively, in the S- and C-band regions with Rogers4450B. The design exhibits a single-negative metamaterial (SNG). The wide band and absorption of the FR-4 are better than those of the Rogers4450B. The polarization angles θ and φ moved in the direction of the E-field and H-field. The E-field and H-field concentrations and current distribution were shown. The proposed unit cell was applied on 1 × 2 and 2 × 2 arrays, and the results were 96.88% and 95.69%, respectively. The absorption of the proposed unit cell is around 99.94%, which is high and more promising than that of some of the recent papers with which it is compared in Table 1. The sensing performance was analyzed for microwave diathermy. These properties indicate that the described MA can be widely implemented for S-band applications.

## Figures and Tables

**Table 1:** Comparison of the high-absorption S-band absorber with those featured in published papers.

Ref. #	Size (mm)	Design Structure	Proposed Band	Absorption (%)	Publishing Year
Hoque et al. [15]	20 × 20	Ring C-shaped	Ku	99.6	2018
Rasheduzzaman Sifat [34]	8 × 8	Modified C-shaped	S-band	Not mentioned	2017
Md.Mehedi Hasan [35]	8 × 10	Reformed I-shaped	S-, C-, and Ku-band	Not mentioned	2017
Mohammad Jakir Hossain [36]	12 × 12	Double C-shaped	S-, C-, X-band	Not mentioned	2017
M.F Zafar et al. [44]	8 × 8	L-shaped	X-band	90	2021
Kollatou et al. [45]	8 × 8	Modified square	X-band	95.81	2013
Borhan et al. [46]	12 × 12	O-shaped	X-band	98.90	2016
Prakash Ranjan [47]	9 × 9	Square-shaped	X-band	99.92	2019
Mohamood et al. [48]	16 × 16	S-shaped	X-band	90.00	2017
Bruno de Araujo [49]	Not mension	V-shaped	Ku	99.8	2020
Zhi Weng [50]	0.00067 × 0.00144	Circular-shaped	Optical Range	99	2019
Ke Bi [51]	9000 × 9000	Square-shaped	Terahertz	Not mentioned	2019
Ke Bi [52]	2 × 2 × 1	Cube/Rectangular-shaped	Terahertz	Not mentioned	2021
Jianchun Xu [53]	12 × 12	Circular/rectangular-shaped	C-, X-band	Not mentioned	2019
Yunsheng Guo [54]	2 × 2 × 2	Cube-shaped	Ku-band	Tunable Absorption up to 100%	2017
Proposed paper	20 × 20	Rectangular-shaped	S-band	99.94	2022

**Table 2:** Permittivity and conductivity of skin, fat, and muscle thickness, permittivity, and conductivity.

	Thickness(mm)	Permittivity	Conductivity [S/m]
Skin	2	41.982	2.0168
Fat	2	5.2138	0.13497
Muscle	2/3/4	2.2216	51.936

## Data Availability

Data availability will be provided if any interesting part is requested. Otherwise, the data will be stored as per research tools.

## Appendix A

**Table A1:** Permittivity, permeability, and reflective index at S_11_ and S_21_ for FR4 substrate at different polarization angles of θ and φ.

(θ & φ)	Angle	Parameter	Frequency Band	Resonance Frequency (GHz)	ε	µ	η	Absorption (%)	Reflection (%)
θ	15°	S_11_	S	3.1	−0.364	0.402	0.001	99.94	0.06
θ	30°	S_11_	S	3.1	−1.506	1.538	0.001	99.89	0.1
θ	45°	S_11_	S	3.1	1.12	−1.18	0.002	999.89	0.01
θ	60°	S_11_	S	3.1	1.12	−1.18	0.002	99.89	0.1
θ	75°	S_11_	S	3.1	1.12	−1.18	0.002	99.89	0.1
θ	90°	S_11_	S	3.1	1.12	−1.18	0.002	99.89	0.1
	0°	S_11_	S	3.1	−0.364	0.402	0.001	99.94	0.06
φ	15°	S_11_	S	3.1	−0.364	0.402	0.002	99.94	0.06
φ	30°	S_11_	S	3.1	−0.364	0.402	0.002	99.94	0.06
φ	45°	S_11_	S	3.1	−0.364	0.402	0.002	99.94	0.06
φ	60°	S_11_	S	3.1	−0.364	0.402	0.002	99.94	0.06
φ	75°	S_11_	S	3.1	−0.364	0.402	0.002	99.94	0.06
φ	90°	S_11_	S	3.1	−0.365	0.402	0.002	99.94	0.06
θ	15°	S_21_	S	1.95	−23.845	42.08	−0.00001	0.3	99.68
		S_21_		3.49	0.904	−286.47	−0.0008	8.1	91.9
		S_21_	C	4.155	4.244	−50.389	−0.0001	6.8	93.15
θ	30°	S_21_	S	1.95	−23.83	42.107	−0.0001	0.7	99.28
		S_21_		3.5	0.952	−271.74	−0.0009	0.8	91.45
		S_21_	C	4.155	4.406	−48.616	−0.0001	0.5	94.40
θ	45°	S_21_	S	1.95	23.833	42.107	−0.0001	0.7	99.28
		S_21_		3.49	0.952	−271.74	−0.0009	8.5	91.45
		S_21_	C	4.155	4.406	−48.616	−0.0001	5.6	94.40
θ	60°	S_21_	S	1.95	−23.833	42.107	−0.0001	0.7	99.23
		S_21_		3.49	0.952	−271.74	−0.0009	8	91.45
		S_21_	C	4.155	4.406	−48.616	−0.0001	5	94.40
θ	75°	S_21_	S	1.95	−23.833	42.107	−0.0001	0.6	99.23
		S_21_		3.49	0.952	−271.74	−0.0009	8.5	91.45
		S_21_	C	4.155	4.406	−48.616	−0.0001	5.6	94.40
θ	90°	S_21_	S	1.95	−23.833	42.107	−0.0001	0.7	99.28
		S_21_		3.49	0.952	−271.74	−0.0009	8.5	91.45
		S_21_	C	4.155	4.406	−42.523	−0.00001	5.6	94.39
	0°	S_21_	S	1.95	−22.734	42.796	−0.00001	0.3	99.68
		S_21_		3.49	0.741	−330.83	−0.001	7	92.03
		S_21_	C	4.155	4.303	−49.603	−0.0001	6	93.35
φ	15°	S_21_	S	1.95	−22.733	42.797	−0.00001	0.3	99.68
		S_21_		3.49	0.904	−286.48	−0.0008	8.1	91.89
		S_21_	C	4.155	4.360	−48.845	−0.0001	6.4	93.54
φ	30°	S_21_	S	1.95	−23.844	42.797	−0.00001	0.3	99.68
		S_21_		3.49	0.904	−286.47	−0.0008	8.1	91.89
		S_21_	C	4.155	4.244	−50.389	−0.0013	6.8	93.15
φ	45°	S_21_	S	1.95	−22.734	42.796	−0.00001	0.3	99.68
		S_21_		3.49	0.904	−286.48	−0.0008	8.1	91.89
		S_21_	C	4.155	4.244	−50.389	−0.0001	6.8	93.15
φ	60°	S_21_	S	1.95	−22.734	42.796	−0.00001	0.3	99.68
		S_21_		3.49	0.904	−286.47	−0.0008	8.1	91.89
		S_21_	C	4.155	4.244	−50.389	−0.0001	6.8	93.15
φ	75°	S_21_	S	1.95	−22.734	42.087	−0.00001	0.3	99.68
		S_21_		3.49	0.904	−286.48	−0.0008	8.1	91.89
		S_21_	C	4.155	4.244	−50.389	−0.0001	6.8	93.15
φ	90°	S_21_	S	1.95	−22.734	42.796	0.00001	0.3	99.68
		S_21_		3.49	0.904	−286.47	−0.0008	8.1	91.89
		S_21_	C	4.155	4.244	−50.389	−0.0001	6.8	93.15

**Table A2:** Permittivity, permeability, and reflective index at S_11_ and S_21_ of Rogers4450B substrate at different polarization angles of θ and φ.

θ & φ	Parameter	Angle	Frequency Band	Resonance Frequency (GHz)	ε	µ	η	Absorption (%)	Reflection (%)
θ	S_11_	15°	S	1.44	−9.0441	13.8534	0.0961	97.16	2.8
				3.96	2.0001	−1.7139	−0.0015	99.49	0.51
			C	4.205	−2.5891	2.0718	−0.0019	99.06	0.94
				5.025	5.2289	−6.1224	−0.0076	93.79	0.62
θ	S_11_	30°	S	1.44	−8.965	13.5525	0.0960	97.28	2.8
				3.96	1.9858	−1.7044	−0.0015	99.50	0.51
			C	4.205	−2.5797	2.0622	−0.0019	99.06	0.94
				5.025	5.2297	−6.1233	−0.0076	93.79	0.62
θ	S_11_	45°	S	1.44	−9.0442	13.8542	0.0961	97.16	2.8
				3.96	2.0032	−1.7161	−0.0015	99.49	0.51
			C	4.205	−2.59	2.0731	−0.0019	99.06	0.94
				5.025	5.2287	−6.1222	−0.0076	93.79	0.62
θ	S_11_	60°	S	1.44	−90438	13.8538	0.0961	97.16	2.8
				3.96	2.0273	−1.7366	−0.0015	99.48	0.51
			C	4.205	−2.6219	2.0969	−0.0019	99.04	0.94
				5.025	5.2295	−6.1243	−0.0076	93.79	0.62
θ	S_11_	75°	S	1.44	−9.0439	13.8539	0.0961	97.16	2.83
				3.96	−2.0134	−1.7259	−0.0015	99.48	0.5
			C	4.205	−2.592	2.0735	−0.0019	99.06	0.9
				5.025	5.2299	−6.124	−0.0076	93.79	0.6
θ	S_11_	90°	S	1.44	−9.0445	13.8543	0.0961	97.16	2.8
				3.96	2.003	−1.7165	−0.0015	99.45	0.5
			C	4.205	−2.5916	2.0738	−0.0018	99.06	0.94
				5.025	5.229	−6.1226	−0.0076	93.79	0.62
	S_11_	0°	S	1.44	−6.429	6.748	0.107	99.382	0.6
				3.96	2.039	−2.331	−0.0019	99.383	0.6
			C	4.205	−6.293	3.555	−0.0021	92.91	4.8
				5.025	5.432	−6.510	−0.008	95.17	7.1
φ	S_11_	15°	S	1.44	−9.044	13.8538	0.0961	97.16	2.8
				3.96	1.9998	−1.7132	−0.0015	99.45	0.51
			C	4.205	−2.5892	2.0728	−0.0019	99.06	0.9
				5.025	5.2302	−6.1238	−0.0076	93.79	6.2
φ	S_11_	30°	S	1.44	−9.0441	13.8535	0.0961	97.16	2.8
				3.96	2.0009	−1.7146	−0.0015	99.49	0.51
			C	4.205	−2.5888	2.0719	−0.0019	99.06	0.9
				5.025	5.2286	−6.122	−0.0076	93.79	6.2
φ	S_11_	45°	S	1.44	−9.044	13.8537	0.0096	97.16	2.8
				3.96	2	−1.7136	−0.0015	99.49	0.51
			C	4.205	−2.5894	2.0724	−0.0019	99.06	0.9
				5.025	5.2291	−6.1221	−0.0076	93.79	6.2
φ	S_11_	60°	S	1.44	−9.044	13.8537	0.0961	97.16	2.8
				3.96	1.9998	−1.7137	−0.0015	99.49	0.51
			C	4.205	−2.5893	2.0719	−0.0019	99.06	0.9
				5.025	5.2289	−6.1223	−0.0076	93.79	6.2
φ	S_11_	75°	S	1.44	−9.0442	13.8534	0.0961	97.16	2.8
				3.96	2.0004	−1.7139	−0.0015	99.49	0.51
			C	4.205	−2.5892	2.0727	−0.0019	99.06	0.9
				5.025	5.2288	−6.1222	−0.0076	93.79	6.2
φ	S_11_	90°	S	1.44	−9.0479	13.8385	0.0964	97.17	2.8
				3.96	1.9979	−1.713	−0.0015	99.49	0.51
			C	4.205	−2.5849	2.068	−0.0019	99.06	0.9
				5.025	5.2284	−6.122	−0.0076	93.79	6.2
θ	S_21_	15°	S	2.05	−19.501	45.14	−0.00001	0.09	99.9
				3.735	2.696	−98.251	−0.00001	2.04	97.95
			C	4.4	5.788	−33.012	−0.00001	1.4	98.40
θ	S_21_	30°	S	2.05	−19.50	45.140	−0.00001	0.09	99.9
				3.735	2.696	−98.276	−0.00001	2.04	97.96
			C	4.4	5.788	−33.012	−0.00001	1.4	98.60
θ	S_21_	45°	S	2.05	−19.50	45.141	−0.00001	0.09	99.9
				3.735	2.697	−98.246	−0.00001	2.04	97.95
			C	4.4	5.788	−33.011	−0.00001	1.4	98.60
θ	S_21_	60°	S	2.05	−19.50	45.141	−0.00001	0.09	99.9
				3.735	2.70	−98.22	−0.00001	2.04	97.96
			C	4.4	5.788	−33.012	−0.00001	1.4	98.59
θ	S_21_	75°	S	2.05	−19.501	45.142	−0.00001	0.09	99.91
				3.735	2.697	−98.232	−0.00001	2.04	97.59
			C	4.4	5.79	−33.01	−0.00001	1.4	98.97
θ	S_21_	90°	S	2.05	−19.501	45.141	−0.00001	0.09	99.91
				3.735	2.697	−98.247	−0.00001	2.04	97.53
			C	4.4	5.788	−33.012	−0.00001	1.4	98.67
	S_21_	0°	S	2.05	−19.54	45.05	−0.00001	0.09	99.91
				3.735	2.60	−101.86	−0.00001	1.8	98.21
			C	4.4	5.35	−35.69	−0.00001	2.1	97.78
φ	S_21_	15°	S	2.05	−19.501	45.141	−0.00001	0.09	99.91
				3.735	2.696	−98.25	−0.00001	2.04	97.95
			C	4.4	5.788	−33.01	−0.00001	1.4	98.59
φ	S_21_	30°	S	2.05	−19.50	45.141	−0.00001	0.09	99.91
				3.735	2.696	−98.25	−0.00001	2.04	97.95
			C	4.4	5.788	−33.012	−0.00001	1.4	98.60
φ	S_21_	45°	S	2.05	−19.50	45.141	−0.00001	0.09	99.91
				3.735	2.696	−98.25	−0.00001	2.04	97.95
			C	4.4	5.788	−33.012	−0.00001	1.4	98.60
φ	S_21_	60°	S	2.05	−19.501	45.141	−0.00001	0.09	99.91
				3.735	2.696	−98.253	−0.00001	2.04	97.95
			C	4.4	5.788	−33.012	−0.00001	1.4	98.60
φ	S_21_	75°	S	2.05	−19.501	45.14	−0.00001	0.09	99.91
				3.735	2.696	−98.25	−0.00001	2.04	97.95
			C	4.4	5.788	−33.012	−0.00001	1.4	98.60
φ	S_21_	90°	S	2.05	−19.50	45.14	−0.00001	0.09	99.91
				3.735	2.70	−98.26	−0.00001	2.04	97.96
			C	4.4	5.788	−33.012	−0.00001	1.4	98.60
